# Dual deficiency of melatonin and dihydrotestosterone promotes stromal cell damage and mediates prostatitis via the cGAS-STING pathway in sleep-deprived mice

**DOI:** 10.1186/s12964-024-01554-5

**Published:** 2024-03-15

**Authors:** Jia Chen, Wenming Ma, Shaoyu Yue, Dongsheng Li, Lei Chen, Cheng Zhang, Yu Guan, Chun Li, Changqin Jiang, Guiyi Liao, Chaozhao Liang, Hui Wang, Sheng Tai

**Affiliations:** Department of Urology, The First Affiliated Hospital of Anhui Medical University, Institute of Urology, Anhui Medical University, Anhui Province Key Laboratory of Genitourinary Diseases, Hefei, 230022 P.R. China

**Keywords:** Sleep deprivation, Melatonin, Dihydrotestosterone, cGAS-STING, Prostatitis

## Abstract

**Purpose:**

Prostatitis is a highly prevalent condition that seriously affects men’s physical and mental health. Although epidemiological investigations have provided evidence of a correlation between insufficient sleep and prostatitis, the pathogenesis of prostatitis remains unclear. We sought to identify the underlying mechanism involved and identify a promising therapeutic target.

**Methods:**

Sleep deprivation (SD) was utilized to establish a mouse model of insufficient sleep in a special device. Prostatitis was observed at different time points post-SD. The degree of prostatitis was evaluated by pathological section and behavioural tests. Using immunofluorescence, western blot, and proteomic analyses, the underlying mechanism of SD-related prostatitis was investigated, and the development and therapeutic target of prostatitis were elucidated.

**Results:**

SD, as an initial pathological trigger, resulted in a reduction in dihydrotestosterone and melatonin levels. Proteomic analysis revealed that the cGAS-STING pathway may play a significant role in inducing prostatitis. The subsequent results illustrated that the dual reduction in dihydrotestosterone and melatonin led to an accumulation of reactive oxygen species and the release of mitochondrial DNA (mt-DNA). The accumulation of mt-DNA activated the cGAS-STING pathway, which recruited inflammatory cells into the prostatic stroma through the secretion of interferon-β. Consequently, an inflammatory microenvironment was formed, ultimately promoting the development of prostatitis. Notably, mice with SD-induced prostatitis gradually recovered to a normal state within 7 days of recovery sleep. However, after being subjected to SD again, these mice tended to have a more pronounced manifestation of prostatitis within a shorter timeframe, which suggested that prostatitis is prone to relapse.

**Conclusions:**

The cGAS-STING pathway activated by dual deficiency of dihydrotestosterone and melatonin plays a comprehensive inflammatory role in SD-related prostatitis. This research provides valuable insights into the pathogenesis, therapeutic targets, and prevention strategies of prostatitis.

**Supplementary Information:**

The online version contains supplementary material available at 10.1186/s12964-024-01554-5.

## Background

The prostate gland is the hormone-dependent gland of the male reproductive system and is primarily regulated by dihydrotestosterone (DHT) [1, 2]. In addition, the prostate is an integral part of the urinary tract (prostatic urethra), bridging the urinary and reproductive systems. Given that the prostate has a unique anatomic location in the genitourinary system, it is easily affected by various pathogenic factors, ultimately leading to prostatitis. Prostatitis is a highly prevalent condition in men, with approximately eight million outpatient visits each year in America [3]. Approximately 35–50% of men are reported to be repeatedly influenced by symptoms induced by prostatitis at some point in their life, including pelvic pain and lower urinary tract symptoms (urinary frequency, urgency, nocturia, urinary hesitancy, weak urinary stream, straining to void and so on), which seriously affects the physical and mental health of patients. The National Institutes of Health (NIH) classification system identifies four types of prostatitis, type III prostatitis, also named chronic prostatitis/chronic pelvic pain syndrome (CP/CPPS), which accounts for approximately 95% of all prostatitis cases [4]. However, the aetiology and pathogenesis of CP/CPPS are still unknown.

Although an epidemiological link between CP/CPPS and several risk factors, such as a sedentary lifestyle, smoking, spicy food, alcohol, neuropsychological stress and sleep disorders, has been reported, the potential mechanism of the pathogenesis and development of CP/CPPS remains unclear [5–7]. Recently, we found that sleep disturbances (prolonged sleep deprivation) were associated with lower urinary tract symptoms, which are typical clinical symptoms of type III prostatitis. Moreover, insufficient sleep has been confirmed to be a critical risk factor for neuroinflammation, enteritis and vascular inflammation, and studies have shown that elevated concentrations of proinflammatory cytokines and autoimmunity are found in men suffering from CP/CPPS and in animal models [8–12]. It has gradually been recognized that autoimmune aetiology plays a vital role in the pathogenesis of CP/CPPS. However, the relationships between sleep disorders and immune regulation and prostatitis and the underlying mechanism need to be further explored [13–15].

Inflammation is typically triggered by the engagement of pattern recognition receptors, which are essential components of the innate immune system responsible for recognizing and responding to danger and damage [16, 17]. Pathogen- and damage-associated molecular patterns (infection, cellular stress, cell damage) released by inflammatory cells activate pattern recognition receptors and cause inflammation. Recently, several studies have revealed that the cGAS-STING signalling pathway is a critical mediator of inflammation in the setting of infection, cellular stress, tissue damage and other conditions [18–21]. The cGAS-STING pathway is a sensor for the accumulation of extranuclear DNA generated by microbial pathogens, self-DNA from the damaged nucleus and mitochondria, in which the activated cGAS-STING pathway continuously produces inflammatory factors to shape the inflammatory microenvironment [18, 22]. In noninfectious diseases, disturbance of the redox balance leads to increased reactive oxygen species (ROS), damage to mitochondria and leakage of mitochondrial DNA (mt-DNA), which is a crucial mechanism for activating the cGAS-STING pathway [23, 24]. Excess activation of the cGAS-STING pathway has been verified to be associated with autoinflammatory and autoimmune diseases, including Aicardi-Goutiéres syndrome, rheumatoid arthritis and systemic lupus erythematosus [25–28]. Additionally, studies have shown that melatonin may normalize cGAS-STING signalling by eliminating excessive cytosolic DNA induced by cell stress, and knockdown of cGAS-STING abrogates melatonin-mediated beneficial effects on mitophagy, cell survival and cardiac function, which indicates the obligatory role of cGAS-STING signalling in melatonin-mediated protection [29, 30]. Notably, insufficient sleep can downregulate the serum level of melatonin, leading to decreased sleep time and prolonged sleep latency [31]. Overall, we hypothesized that insufficient sleep may induce prostatitis by regulating melatonin-mediated activation of the cGAS-STING pathway.

**Fig. 1:**
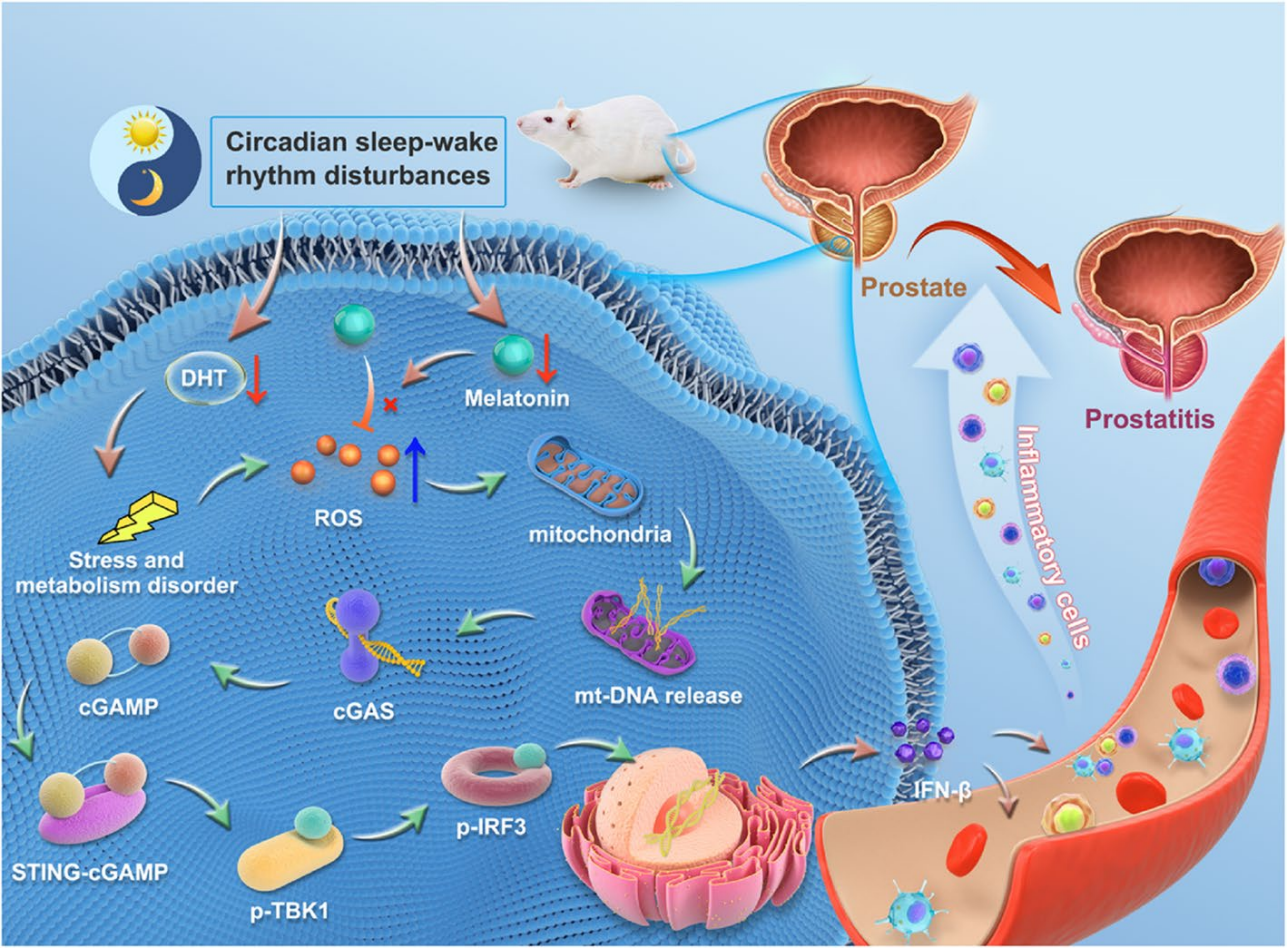
Effect of sleep deprivation (SD) on prostate in ICR mice. SD results in the simultaneous reduction of melatonin (MT) and dihydrotestosterone (DHT), which leads to an accumulation of reactive oxygen species (ROS) and the release of mitochondrial DNA (mt-DNA), activating the cGAS-STING pathway. The activated cGAS-STING pathway in turn recruits inflammatory cells into prostatic stroma and subsequently promotes the development of prostatitis

Here, we showed that simultaneous reductions in melatonin (MT) and DHT disrupted the intracellular redox balance in the prostatic stroma of sleep-deprived mice, resulting in the accumulation of reactive oxygen species in the prostate, which led to mitochondrial damage and thus promoted mt-DNA release from the mitochondria to the cytosol. Correspondingly, the released mt-DNA activated the cGAS-STING pathway, subsequently enhancing the recruitment of inflammatory cells into the prostate, leading to the formation of an inflammatory microenvironment (prostatitis) (Fig. 1). These findings may reveal a considerable pathogenic risk factor for prostatitis and its potential regulatory mechanism, which may lead to accurate therapeutic targets and strategies for treating patients with sleep disorder-associated prostatitis.

## Methods

### Mice and sleep deprivation design

The 6- to 8-week-old male ICR mice used in our study were purchased from the Nanjing Biomedical Research Institute of Nanjing University (Nanjing, China). All animals were housed and maintained under specific pathogen-free conditions at Anhui Medical University animal care facilities.

As previously described [8], chronic sleep deprivation was performed in a transparent cylindrical device 25 cm in height and 30 cm in diameter. At the bottom of the device is a motor-driven metal rod whose speed can be controlled (12 s per turn). Mice were acclimated to the barrel for seven days before sleep deprivation began. Using a smart circuit controller with a timed off function, we controlled the sleep deprivation device to turn off for one hour after every five hours of operation so that the mice would stay awake for 20 h throughout the day. The control mice were fed the same cylindrical device with adequate water and food and normal sleep.

### Behavioural testing

Behavioural testing was based on the concept of cutaneous hyperalgesia resulting from referred visceral pain. Pain responses were assessed by tactile allodynia using von Frey filaments before sleep deprivation (baseline) and 1, 2, 3, 4, 5 and 6 weeks after the start of sleep deprivation. We tested tactile allodynia via the use of von Frey filaments applied to the abdomen near the prostate according to previous methods. Three types of behaviours were considered to be positive responses to von Frey filament stimulation: (1) sharp retraction of the abdomen, (2) immediate licking or scratching of the area of filament stimulation, or (3) jumping.

### Protein extraction and western blot analysis

Proteins were resolved on a polyacrylamide gel and blotted onto a PVDF membrane (Millipore). The membranes were blocked with QuickBlock™ Western buffer (Beyotime, Shanghai, China). The membranes were then incubated with primary antibody overnight at 4 °C, washed, and then hybridized with the appropriate horseradish peroxidase–conjugated secondary antibody (rabbit, Elabscience, Wuhan, China). Detection was performed with an enhanced chemiluminescence kit (Advansta, CA, USA). The following antibodies were used: anti-cGAS (1:1000; CST, #31,659), anti-IRF3 (1:1000; CST, #4302), anti-P-IRF3 (1:500; CST, #4947), anti-TBK1 (1:1000; CST, #3504), anti-P-TBK1 (1:1000; CST, #5483), BMAL1 (1:1000; Affinity, DF10308), SRD5A2 (1:1000; Abcam, ab124877) and anti-GAPDH (1:1000; Affinity, AF7021).

### Histological and immunofluorescence analyses

Haematoxylin and eosin staining was performed on paraffin-embedded tissue. Briefly, tissue section (5 μm) were dewaxed, rehydrated, stained for 4 min with haematoxylin (Merck), shortly washed in distilled water, and incubated for 1 min in eosin solution (Merck). The slides were then mounted using mounting medium (BioMount HM, Bio Optica). The immunofluorescence primary antibody used was CD45 (1:500; Servicebio, GB113886). Based on previous methods [14], the degree of prostatic inflammation was assessed using a score of 0–3 (0: noninflammation; 3: severe inflammatory cell infiltration).

### IHC analysis

The slides of paraffin-embedded tissue specimens were dewaxed, rehydrated, and heated at 100 °C for 10 min in citric acid buffer (0.01 M, pH 6.0) for antigen retrieval. The slides were incubated in 3% hydrogen peroxide solution (10,011,208; Pharmaceutical Group Chemical Reagent Co., Ltd.) for 15 min at room temperature and washed three times in phosphate-buffered saline (PBS; pH 7.4). The slides were blocked with 10% bovine serum albumin. Then, the slides were incubated with an anti-P-IRF3 antibody (1:1,000; Affinity, AF2436) and an anti-P-TBK1 antibody (1:1000; Affinity, AF8190) overnight at 4 °C. After three washes with PBS, the slides were incubated with biotinylated goat antirabbit IgG (1:200) for 2 h at room temperature. Finally, DAB (G1211, Servicebio, China) was used to detect the immune complexes, and the slices were counterstained with haematoxylin. Relative expression levels were analysed with ImageJ software (National Institutes of Health, Bethesda, MD).

### Electron microscopy

The mouse prostate was fixed in 2% glutaraldehyde in 0.1 M sodium cacodylate buffer (pH 7.4) at 4 °C overnight. Fixation was performed with 1% OsO4. The samples were then dehydrated in graded ethanol and embedded in Epon resin. Ultrathin sections were stained with 2% uranyl acetate and examined under a transmission electron microscope (HT7800, HITACHI).

### ELISA

Cytokine levels in mouse plasma and prostate tissue homogenate samples were detected using ELISA kits for MT (E-EL-M0788c, Elabscience, Wuhan, China), DHT (E-EL-0031c, Elabscience, Wuhan, China), E2 (E-OSEL-M0008, Elabscience, Wuhan, China), T (E-OSEL-M0003, Elabscience, Wuhan, China), GnRH (E-EL-0071c, Elabscience, Wuhan, China), LH (E-EL-M3053, Elabscience, Wuhan, China), IFN-β (EK2236-96, MULTISCIENCES, Hangzhou, China), TNF-ɑ (EK282/4–96, MULTISCIENCES, Hangzhou, China), IL-6 (EK206/3–96, MULTISCIENCES, Hangzhou, China), and PGE2 (EK8103/2–96, MULTISCIENCES, Hangzhou, China).

### Cell culture and cell viability assay

The WPMY-1 cell line (a human prostatic stromal myofibroblast line) and RWPE-1 cell line were obtained from Procell (Wuhan, China). The WPMY-1 cell line was maintained in DMEM supplemented with 5% FBS, 100 U/ml penicillin, and 100 µg/ml streptomycin. The RWPE-1 cell line was maintained in KM medium without FBS. All cells were cultured at 37 °C in a humidified environment of 5% CO2 in the air and tested negative for mycoplasma contamination.

### Mitochondrial membrane potential

The mitochondrial membrane potential was analysed using a Mitochondrial Membrane Potential Assay Kit with JC-1 (Beyotime, Shanghai, China). The cells were washed once with PBS and incubated in prewarmed media supplemented with 5 mg/mL JC-1 dye for 30 min at 37 °C in a 5% CO_2_ incubator. The cells were then washed three times with PBS and imaged using an Olympus IX-73. Data acquisition was also performed on a CytoFLEX LX (Beckman Coulter), and the data were analysed by using FlowJo software (version 10.0).

### Cellular ROS assay

A reactive oxygen species assay kit (Beyotime, Shanghai, China) was used to measure the intracellular production of ROS. WPMY-1 cells were washed once with PBS and incubated with fresh media containing 10 µM DCFH-DA for 30 min. The cells were washed three times with PBS, after which the fluorescence of DCFH-DA (excitation of 488 nm and emission of 525 nm) was measured using an Olympus IX-73. We similarly examined ROS levels using flow cytometry.

### mt-DNA assay

The cells were divided into control and MT and DHT double-deficient groups. WPMY-1 cells were seeded into confocal dishes at 5 × 10^3^ cells. The discarded supernatant was washed with serum-free medium three times, and serum-free medium containing PicoGreen (Yeasen, Shanghai, China) and MitoTracker (Beyotime, Shanghai, China) was added and incubated at 37 °C for 30 min. The cells were washed 3 times in serum-free medium. Laser scanning confocal microscopy (LSM800, Zeiss) was used to monitor mitochondrial DNA extravasation into the cytoplasm.

### Statistical analysis

A minimum of four mice were included per experimental group. For western blot and cell culture experiments, a minimum of two biological replicates were performed. For parametric data, the significance of the differences was analysed using one-way ANOVA and an unpaired Student’s *t* test. Prism software version 9 (GraphPad Software, Inc.) was used for all the statistical analyses. All the results are expressed as the means ± SEMs. *P* < 0.05 was considered to indicate statistical significance. n refers to biological replicates that are reported in each figure legend.

## Results

### Prostatitis induction in a time-dependent manner in sleep-deprived mice

**Fig. 2:**
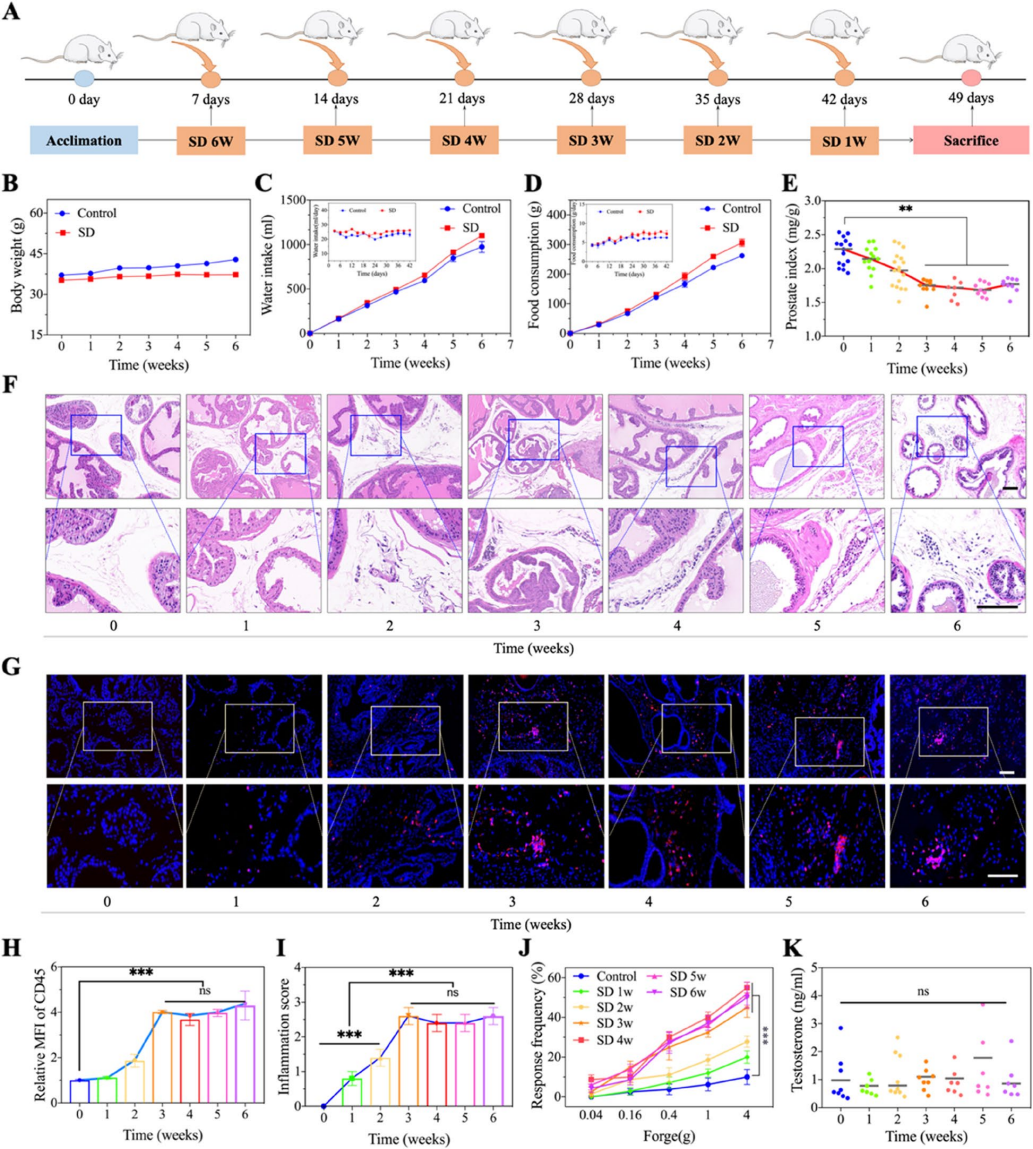
SD-mediated formation of prostatitis in a time-dependent manner. **A** Schematic illustration of SD process in mice for different duration. The start time of SD in ICR mice was different, and but these mice were sacrificed at the same time point. All of mice were kept awake for 20 h a day (4 h of sleep). **B** Changes of body weight in mice during the period of SD. **C-D** Cumulative and daily water intake and food consumption during the period of SD. **E** Evaluation of prostate sizes changes by prostate index in mice after different SD duration. **F** HE staining showing the changes of prostate in mice after different SD duration. Scale bar, 100 μm. **G-H** Immunofluorescence staining of CD45 and corresponding mean fluorescence intensity (MFI) analysis showing the inflammatory cells infiltration in prostate of mice after different SD duration. Scale bar, 100 μm. **I** Inflammation score analysis of prostate in mice after different SD duration. **J** Examination of tactile allodynia by Von Frey filaments in mice after different SD duration. **K** Changes of serous testosterone level in mice during the period of SD. Data were presented as means ± SEM (*n* ≥ 5). Statistical significance was calculated using the one-way ANOVA (**E**, and **H** to **K**). ns, no significance, **P < 0.01, and ***P < 0.001

To determine the association between chronic sleep deprivation (SD) and prostatitis in mice, we divided the mice into three groups, with the mice remaining awake for 22, 20 and 18 h per day for 28 consecutive days based on the chronic sleep deprivation protocol. Abnormal changes in the prostate were not observed in the mice that were awake for 18 h per day, but hyperemia and the dilatation of blood vessels, perivascular infiltration of inflammatory cells and epithelial structural disorders were found in the prostate of mice that were awake for 22 and 20 h per day, which illustrated the occurrence of pathological changes in the prostate, consistent with the characteristics of prostatitis (Fig. S1). Based on these findings, we also investigated the changes in the prostate in mice that slept for 4 h per day (awake for 20 h) as the number of SD days increased (1 to 6 weeks) (Fig. 2A). During the period of sleep deprivation, the potential changes in body weight due to SD were evaluated, which is a simple, intuitive and effective index for identifying systemic influences. As shown in Fig. 2B, compared with that in the normal group, there was slight body weight loss in the SD group from 1 week to 6 weeks. Body weight loss was time dependent, with the largest difference occurring between the normal group (42.87 ± 0.41 g) and the SD group (37.26 ± 0.82 g) at the 6th week. However, the water intake and food consumption of the SD group were greater than those of the control group (Fig. 2C,  D), indicating not only that SD had no effect on water or food intake but also that weight loss in mice in the SD group was not associated with water or food consumption. Next, we focused on the effects of SD on the prostate. First, the prostate was dissected from the mice, and gross prostate specimens were observed. The results revealed that the prostate size (prostate index, prostate weight/body weight) decreased as the duration of SD increased from 1 to 3 weeks. While the duration of SD continued to increase, the size of the prostate tended to increase, starting from the third week after SD (Fig. 2E).

Subsequently, histological changes in the prostate of sleep-deprived mice were evaluated. Representative characteristics of prostatitis were observed, and the degree of pathological changes also increased in a time-dependent manner (Fig. 2F). The number of inflammatory leukocytes, marked by CD45 (red) and leukocyte common antigen, increased during the SD from 1 to 6 weeks, and the number of CD45^+^ cells tended to level off, starting from the third week (Fig. 2G, H). Furthermore, the degree of inflammation, which was assessed by a classical scale (0 to 3 points), and the degree of pelvic pain induced by prostatitis were utilized to evaluate prostatitis; these findings confirmed that the changes in the prostate stabilized after three weeks of SD (Fig. 2I, J). The prostate gland requires hormones for proliferation and maintenance of cellular viability. Therefore, histological examination (HE staining) of testicles that produced sex hormones was applied to evaluate the underlying effects on testicles, and no significant pathological changes were found (Fig. S2). Furthermore, we analysed the levels of several hormones, including testosterone (T), oestradiol (E2), luteinizing hormone (LH), and gonadotropin-releasing hormone (GnRH), in normal mice and sleep-deprived mice. As a result, there was no significant change in the expression of these hormones either inside the serum or inside the prostate tissue (Figs. 2K and S3).

### Prostatitis and proteomic analysis in sleep-deprived mice

**Fig. 3:**
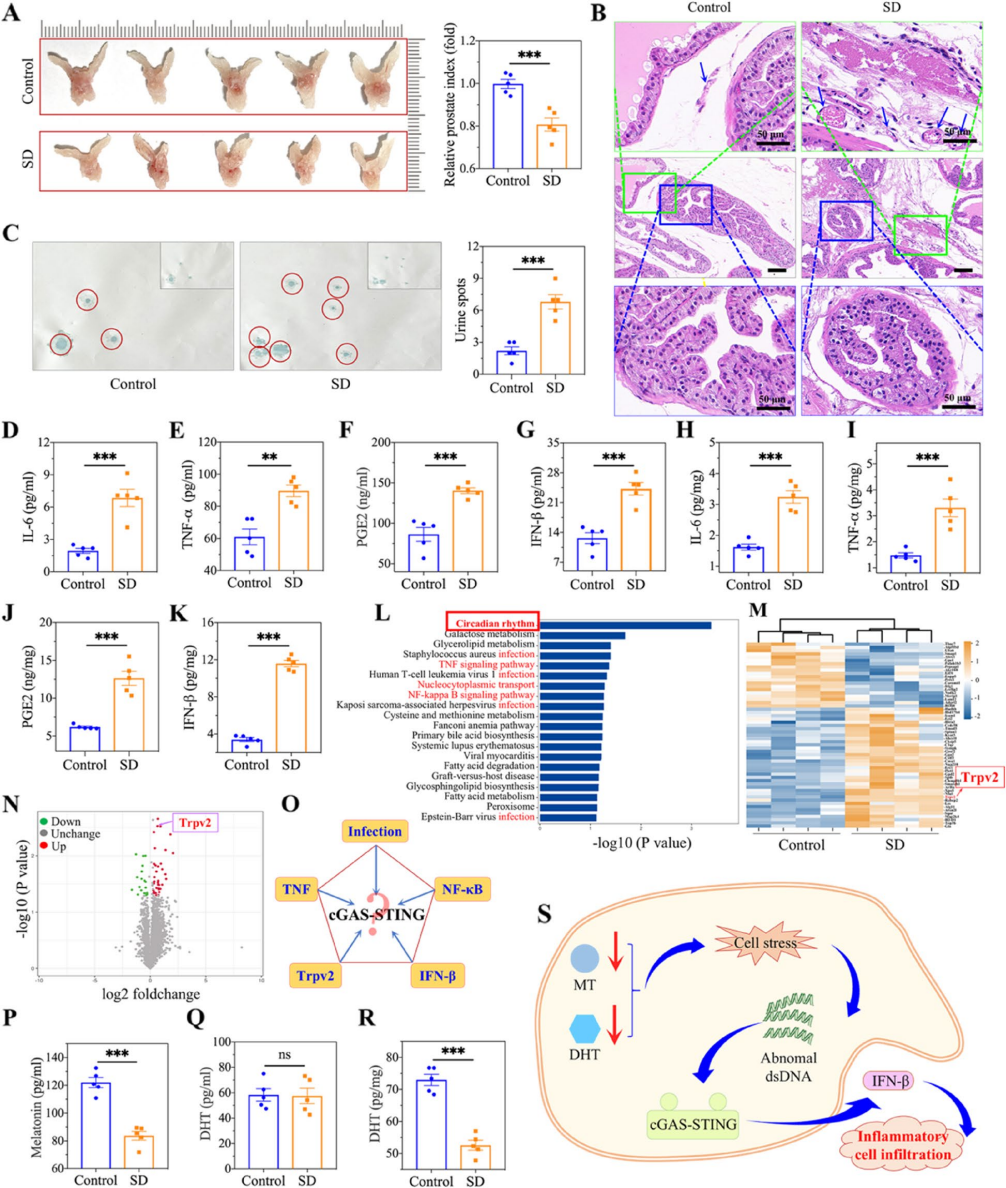
Characteristics and proteomic analysis of prostate in mice after 3 weeks of SD. **A** Photos of prostate and corresponding sizes (prostate index) analysis. **B** HE staining showing the typical pathological changes of prostatitis. **C** Quantification of urinary frequency by urine spots assay. **D-G** ELISA analysis of cytokines in serum, including IL-6, TNF-α, PGE2 and IFN-β. **H-K** ELISA analysis of cytokines in prostate tissue, including IL-6, TNF-α, PGE2 and IFN-β. Proteomic analysis of control and SD mice including (**L**) KEGG pathway enrichment, (**M**) hierarchical clustering heatmap, and (**N**) volcano plot of differentially expressed proteins. **O** Proteomic analysis showing the underlying mechanism of prostatitis induction. **P** Analysis of MT level in serum after 3 weeks of SD. **Q-R** Analysis of DHT level in serum and prostate tissue after 3 weeks of SD. **S** Schematic hypothesis of the cGAS-STING pathway activation by the dual deficiency of MT and DHT for inducing inflammatory microenvironment in prostate. Data were presented as means ± SEM (*n* = 5). Statistical significance was calculated using the Student’s *t* test (**A**, **C**, **D**-**K**, and **P**-**R**). ns, no significance, **P < 0.01, and ***P < 0.001

As shown above, prostate inflammation was induced, and the extent of inflammation plateaued approximately 3 weeks after SD, as determined by histological assessment and inflammatory index examination. To further explore the potential mechanism of prostate inflammation induced by SD, we used three weeks of SD as the time point at which the animal prostatitis model was established. As a result, we observed that the volume of the mouse prostate decreased by the end of the 3-week SD period (Fig. 3A). The prostate index was used to quantitatively determine the change in prostate volume. Compared with that in normal mice, the prostate volume in sleep-deprived mice was reduce by 20% (Fig. 3A). Moreover, we noted several noticeable pathological changes in the sleep-deprived prostate by histological examination (HE staining), including vacuolization of the prostate epithelium, congestion of the interstitial blood vessels, fibrosis and infiltration of inflammatory cells (Fig. 3B). Based on the abnormal changes in prostatitis, the sleep-deprived mice presented a decreased urinary frequency, which was identified by the number of urine droplets per hour (urine spots). There were significantly more urine spots in sleep-deprived mice (7.33 ± 0.88) than in the normal group (2.67 ± 0.33) (Fig. 3C). Additionally, inflammatory mediators secreted by inflammatory cells were measured to further reveal prostatitis. As shown in Fig. 3D-G, compared with those in the control group, the serum levels of IL-6, TNF-ɑ, PGE2 and IFN-β in the SD group were obviously increased. Similarly, significant differences in these indicators between the control group and the SD group were observed in prostate tissue. In particular, the level of IFN-β in the SD group (11.59 ± 0.35 pg/mg) was 3-fold greater than that in the control group (3.40 ± 0.22 pg/mg) (Fig. 3H-K), which suggested that IFN-β could be a key effector that potentially mediates inflammation in the prostate in sleep-deprived mice.

To investigate the underlying mechanism causing the alterations in prostatitis, prostate tissues were evaluated via proteomic analysis and KEGG pathway enrichment of the proteome data. As a result, circadian rhythm, the TNF signalling pathway, the virus infection pathway, and the NF-kappa B signalling pathway were enriched according to the KEGG analysis (Fig. 3L) and the differential protein expression between the control and SD groups (Fig. 3M, N). These enriched pathways and differential proteins (Trpv2) were previously reported to be associated with the cGAS-STING pathway [32–34], which plays a crucial role in inflammation (Fig. 3O). Hence, we suspected that the cGAS-STING pathway is involved in the process of mediating prostatic inflammation in sleep-deprived mice. The circadian rhythm signalling pathway was enriched according to the KEGG analysis. MT, a free radical scavenger, is the hormone most susceptible to circadian rhythms. As shown in Fig. 3P, the MT levels in the SD group (83.64 ± 3.20 pg/ml) were significantly lower than those in the control group (122.01 ± 3.66 pg/ml), which illustrated the reduced antioxidant capacity of the sleep-deprived mice. In addition, in contrast to testosterone, DHT, an active metabolic product of the conversion of testosterone by 5α-reductase, is essential for prostate development and homeostasis. Therefore, we assayed the levels of DHT in serum and prostate tissue. The results showed that DHT levels in the serum remained unchanged, while DHT levels decreased in prostate tissue in the SD group (Fig. 3Q, R). The main source of DHT in prostate is conversion from testosterone via type 2 of 5α-reductase (SRD5A2). The decreased level of SRD5A2 in prostate was found, which may be an key reason for the decline in DHT (Fig. S4). The A decreased DHT level disrupts mitochondrial aerobic metabolism, increasing oxidative stress in the prostate [35]. On the basis of the findings above, we propose that dual deficiency of MT and DHT in prostate tissue stimulates the cGAS-STING pathway by disrupting the redox balance to recruit inflammatory cells into the prostate after SD (Fig. 3S).

### cGAS-STING pathway activation is induced by MT and DHT deficiency in WPMY-1 cells

**Fig. 4:**
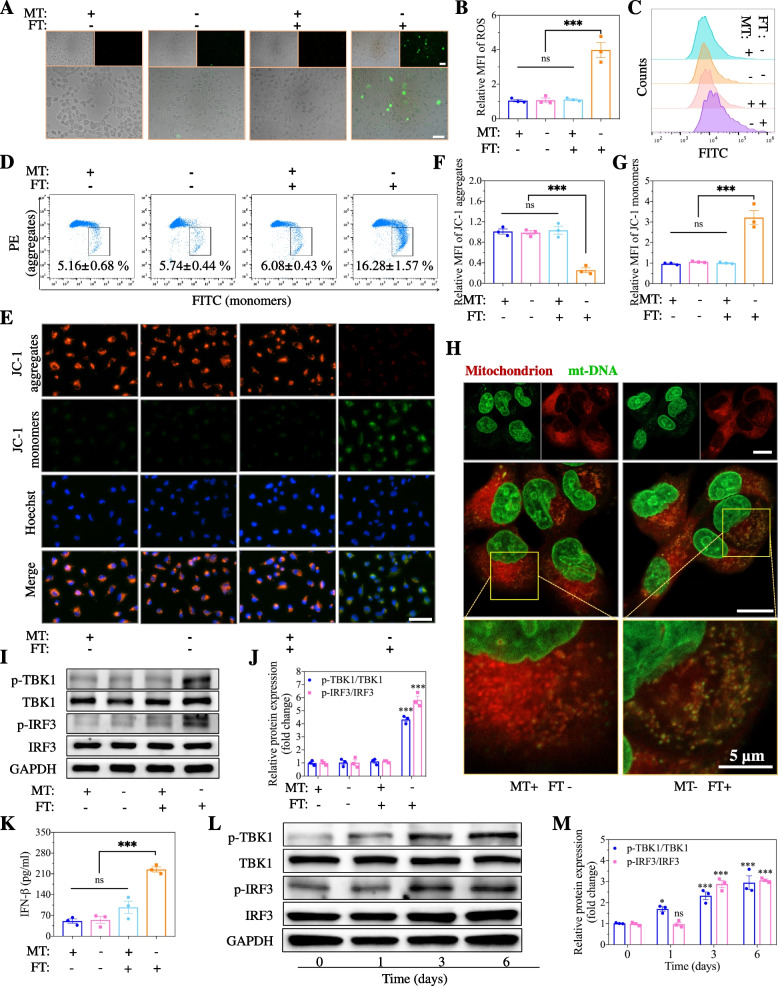
Activation of cGAS-STING pathway by dual deficiency of MT and DHT in WPMY-1 cells. **A-C** Fluorescence images, corresponding mean fluorescence intensity, and flow cytometric analysis of intracellular ROS in WPMY-1 cells after incubation with different concentrations of MT (0 and 100 µM) and FT (0 and 25 µM). Scale bar, 50 μm. **D** Flow cytometric analysis of mitochondrial membrane potentials in WPMY-1 cells after treated by MT (0 and 100 µM) and FT (0 and 25 µM). **E-G** Fluorescence microscope images and quantitative analysis of the mitochondrial membrane potential via the changes of aggregates (PE) and JC-1 monomers (FITC) in WPMY-1 cells. Scale bar, 50 μm. **H** Fluorescence images of mitochondrial DNA (mt-DNA) in cytoplasm of WPMY-1 cells after treated by MT and FT. Scale bar, 10 μm. **I-J** Western blot and corresponding analysis of the essential proteins of cGAS-STING pathway in WPMY-1 cells after incubation with different concentrations of MT (0 and 100 µM) and FT (0 and 25 µM) for 3 days hours. **K** The IFN-β concentration in the culture medium of WPMY-1 cells incubated with with different concentrations of MT (0 and 100 µM) and FT (0 and 25 µM). **L-M** Western blot and corresponding analysis of the essential proteins of cGAS-STING pathway in WPMY-1 cells after incubation with MT (0 µM) and FT (25 µM) for different days. Data were presented as means ± SEM (*n* = 3). Statistical significance was calculated using the one-way ANOVA (**B**, **F**, **G**, **J**, **K** and **M**). ns, no significance, and ***P < 0.001

MT and finasteride (FT, a 5α-reductase inhibitor that inhibits DHT production) were used to establish a model for MT and DHT deficiency. After incubation with MT (0 µM) and finasteride (25 µM) for 3 days, intracellular ROS accumulated in WPMY-1 cells, whereas no ROS accumulation was observed in cells treated with MT or finasteride alone (Fig. 4A, B). The flow cytometry results also provided the same evidence (Fig. 4C). DNA released from damaged mitochondria, which is characterized by changes in mitochondrial membrane potentials, is the key source of endogenous DNA that stimulates the cGAS–STING pathway. Thus, JC-1 staining was utilized to measure the mitochondrial membrane potential of WPMY-1 cells. Similarly, the intracellular damage to mitochondria identified by changes in mitochondrial membrane potential was found only in cells treated with MT (0 µM) or finasteride (25 µM), and this change also occurred in a time-dependent manner (Fig. 4D-G). Subsequently, we used DNA probes to observe the cytoplasmic double-stranded DNA (ds-DNA) that can directly activate the cGAS-STING pathway. Large amounts of abnormal ds-DNA were found in the cytoplasm of cells coincubated with MT (0 µM) and finasteride (25 µM) (Fig. 4H). Therefore, the expression of effector proteins of the cGAS-STING pathway, including TBK1 (p-TBK1) and IRF3 (p-IRF3), was upregulated in cells treated with MT (0 µM) and finasteride (25 µM) (Fig. 4I, J), which induced IFN-β production (Fig. 4K). The expression levels and quantitative analysis of p-TBK1 and p-IRF3 increased with increasing treatment time for MT (0 µM) and finasteride (25 µM) (Fig. 4L, M), which was consistent with the pathological changes in the prostate in vivo and the results shown in Fig. 4D, E. However, no significant changes in the expression levels of p-TBK1 or p-IRF3 in prostate epithelial cells (RWPE-1) were observed, suggesting that prostatic inflammatory changes occurred primarily in the prostate stroma (Fig. S5).

### cGAS-STING pathway activation by ROS-mediated mt-DNA release in sleep-deprived mice

**Fig. 5:**
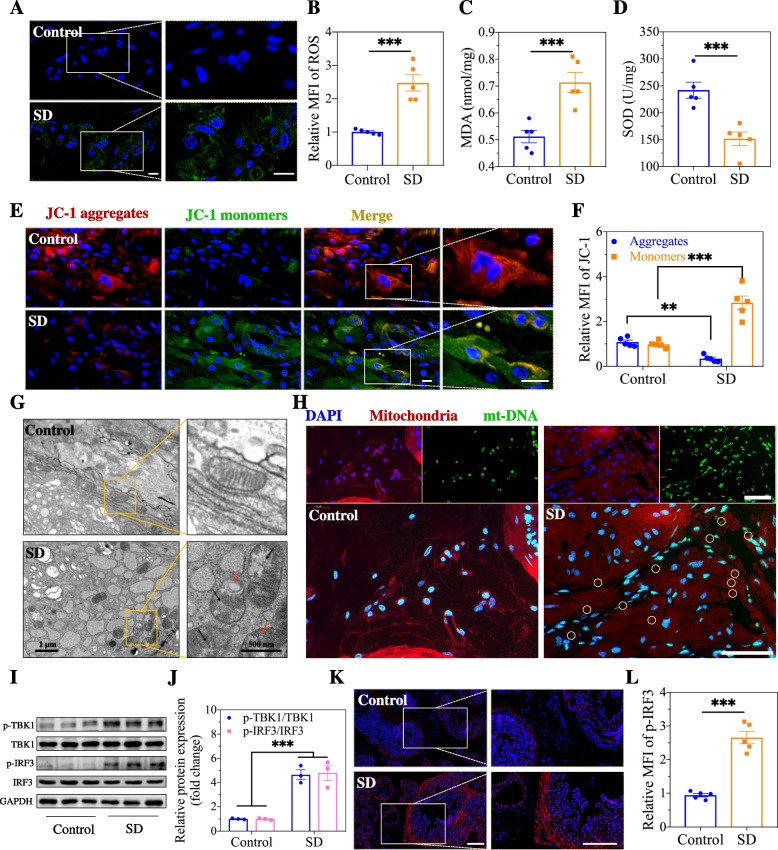
Activation of cGAS-STING pathway by ROS-mediated mt-DNA release in sleep-deprived mice. **A-B** Fluorescence images and corresponding mean fluorescence intensity of intracellular ROS in prostate. Scale bar, 10 μm. **C-D** The indexes of oxidative stress in prostate, including MDA and SOD. **E-F** Fluorescence microscope images and quantitative analysis of the mitochondrial membrane potential via the changes of aggregates (PE) and JC-1 monomers (FITC). Scale bar, 10 μm. **G** Morphology of mitochondria of prostate stromal cells. **H** Fluorescence images of mitochondrial DNA (mt-DNA) in cytoplasm of prostate stromal cells. Scale bar, 50 μm. **I-J** Western blot and corresponding analysis of the essential proteins of cGAS-STING pathway in prostate. **K-L** Immunofluorescence images and quantitative analysis of the p-IRF3 expression in prostate stromal cells. Scale bar, 50 μm. Data were presented as means ± SEM (*n* = 5). Statistical significance was calculated using the Student’s *t* test (**B**-**D**, **F**, **J**, and **L**). **P < 0.01, and ***P < 0.001

In the SD group, we observed an increase in the level of ROS in prostate stromal cells via the use of a fluorescent probe (Fig. 5A, B). A significant accumulation of MDA, an important indicator of lipid peroxidation by free radicals, was found in the prostate tissue of sleep-deprived mice (Fig. 5C). Likewise, superoxide dismutase (SOD), a key marker of oxidative stress, was assayed, and its activity was found to be significantly inhibited in the prostate tissue of sleep-deprived mice (Fig. 5D), indicating increased oxidative stress in the prostate. Afterwards, the mitochondrial membrane potential in prostate stromal cells was evaluated with a JC-1 probe. As a result, loss of JC-1 red fluorescence (a shift from J-aggregates (red) to monomers (green)) was observed, which indicated that the intracellular mitochondrial membrane potential of stromal cells decreased after SD (Fig. 5E, F). Next, the mitochondrial ultrastructure was examined via electron microscopy, and fragmentation, swelling, vacuoles in the mitochondrial matrix, and loss of cristae were observed in prostate stromal cells (Fig. 5G), further illustrating severe mitochondrial damage. Colocalization was visualized with DAPI staining of the nuclear DNA and with ds-DNA staining of the cytoplasmic DNA. In the SD group, we found a large amount of ds-DNA in the cytoplasm, which was the most critical factor in activating the cGAS-STING pathway (Fig. 5H). Subsequently, the expression levels and quantitative analysis of p-TBK1 and p-IRF3 in prostate tissue were assayed. As shown in Fig. 5I, J, compared with those in the control group, we found increased levels of p-TBK1 and p-IRF3 and, accordingly, an increased ratio of p-TBK1 to total TBK1 and a ratio of p-TBK1 to total TBK1 in the SD group, suggesting the activation of cGAS-STING. Furthermore, immunofluorescence staining analysis of changes in location and expression of p-IRF3 revealed that p-IRF3 was obviously upregulated (control vs. SD, 1:2.73) at the protein level in prostate stromal cells after SD, while no significant changes were found in epithelial cells (Fig. 5K, L), which was consistent with the in vitro results (Fig. S5). This is a possible reason why prostatic inflammatory changes are mainly localized in the stroma.

### cGAS-STING pathway activation and prostatitis induction by inhibiting MT and DHT in normal mice

**Fig. 6:**
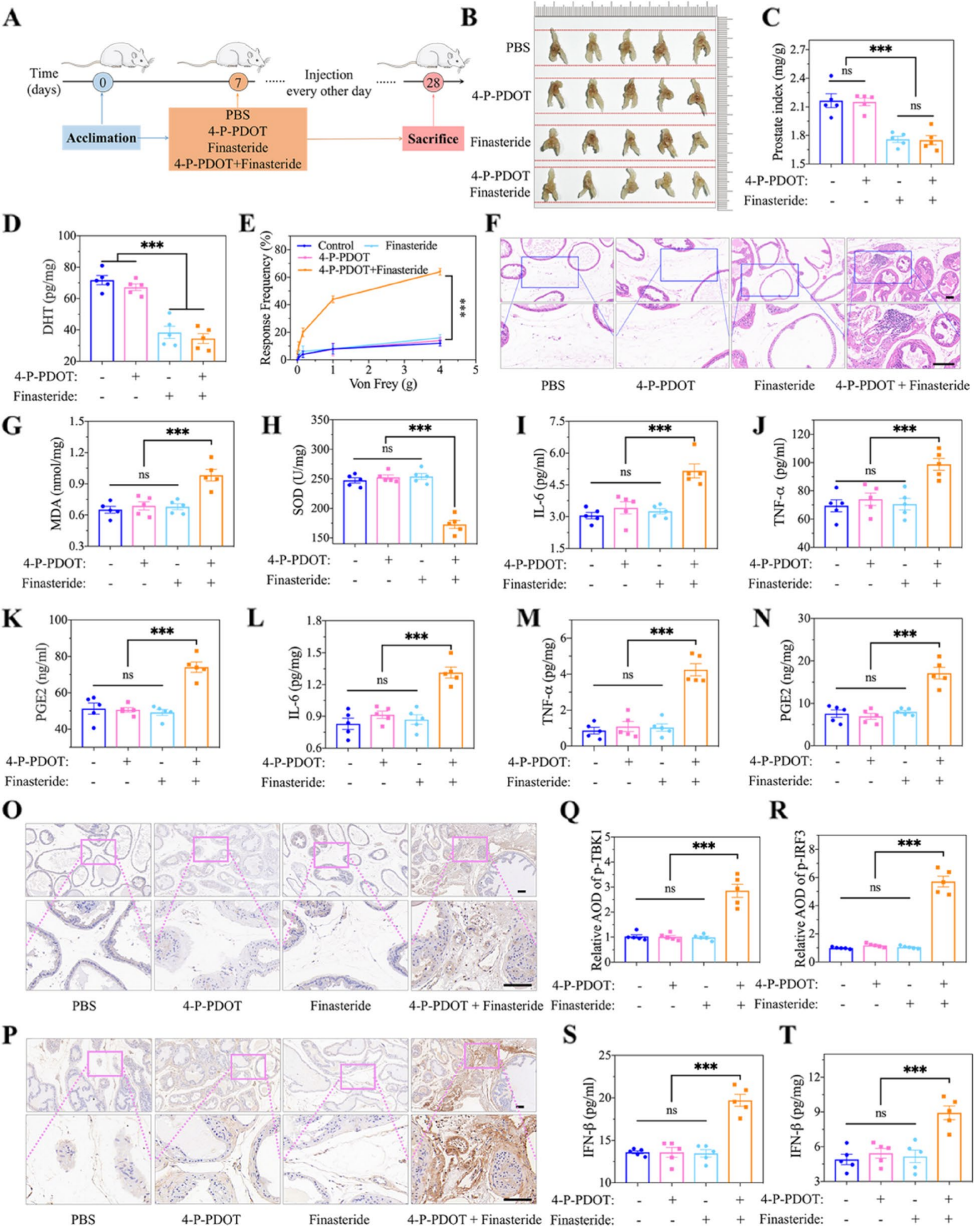
Induction of cGAS-STING pathway-mediated prostatitis by co-inhibiting MT and DHT in normal mice. **A** Schematic illustration of treatment by MT inhibitor (4-P-PDOT) and DHT inhibitor (finasteride) in normal mice for 3 weeks. 4-P-PDOT and finasteride were used to establish the mice model with dual deficient MT and DHT, which was applied to verify the relationship between dual deficient MT and DHT and prostatitis in SD mice. **B-C** Photos of prostate and corresponding sizes (prostate index) analysis. **D** The level of DHT in prostate of mice treated by finasteride. **E** Changes of tactile allodynia by Von Frey filaments. **F** HE staining showing the changes of prostate in mice after 4-P-PDOT and finasteride treatment. Scale bar, 100 μm. **G-H** The indexes of oxidative stress in prostate, including MDA and SOD. **I-K** ELISA analysis of cytokines in serum, including IL-6, TNF-α, and PGE2. **L-N** ELISA analysis of cytokines in prostate tissue, including IL-6, TNF-α, and PGE2. **O-P** Immunohistochemical staining of p-TBK1 and p-IRF3. Scale bar, 100 μm. **Q-R** The corresponding average optical density (AOD) of immunohistochemistry for p-TBK1 and p-IRF3. **S-T** The changes of IFN-β levels in serum and prostate tissue. Data were presented as means ± SEM (*n* = 5). Statistical significance was calculated using the one-way ANOVA (**C-E**, **G-N**, and **Q-T**). ns, no significance, and ***P < 0.001

An MT inhibitor (4-P-PDOT) and a DHT inhibitor (finasteride) were used to establish a model with dual deficiency of MT and DHT in normal mice for 21 days (Fig. 6A), which revealed the effects of MT and DHT on sleep-deprived mice, further verifying the potential mechanism of MT and DHT deficiency in the induction of prostatitis. After administration every other day for three weeks, for group I (PBS), group II (4-P-PDOT), group III (finasteride) and group IV (4-P-PDOT + finasteride), we observed that the prostate volume (prostate index) in groups III and IV (1.76 ± 0.03 and 1.75 ± 0.05, respectively) was lower than that in groups I and II (2.17 ± 0.07 and 2.15 ± 0.04, respectively) (Fig. 6B, C), indirectly indicating that finasteride played an expected role in the reduction in DHT. We also evaluated the DHT concentration in the four groups by ELISA, and the results were consistent with those shown in Fig. 6C. The DHT levels in groups I and II were greater than those in groups III and IV, and no differences were observed between groups I and II between groups III and IV (Fig. 6D). Afterwards, pelvic pain, a classic syndrome of prostatitis, was measured, and the results showed an increased degree of pain in group IV compared with the other three groups (Fig. 6E). Pathologic changes in prostatitis, including vacuolization of the prostate epithelium, congestion of the interstitial blood vessels, fibrosis and infiltration of inflammatory cells, were found by representative HE staining of prostate sections from group IV (Fig. 6F), which verified the correlation between double-deficient MT and DHT and prostate inflammation. In group IV, MDA and SOD, key indicators of oxidative stress, were also tested, and changes in MDA and SOD indicated that the level of oxidative stress was markedly increased in the prostate (Fig. 6G, H). Additionally, we observed an increase in inflammatory cytokines, including IL-6, TNF-ɑ, and PGE2, in either the serum or the prostate tissue in group IV (Fig. 6I-N). The expression of crucial proteins in the cGAS-STING pathway, p-TBK1 and p-IRF3, was detected via immunohistochemistry. As shown in Fig. 6O-R, we noted significantly increased expression of p-TBK1 and p-IRF3 in group IV; these proteins are located in the prostatic stromal region. For the expression of p-IRF3, the average optical density (AOD) was 6-fold greater in group IV than in the other groups. Thus, the level of IFN-β, a product derived from the activation of the cGAS-STING pathway, was greater in the present study than in the other groups, and there was no significant difference among groups I, II and III (Fig. 6S, T). These findings further revealed that sleep-deprived mice lacking both MT and DHT could activate the cGAS-STING pathway to induce prostate inflammation.

### Prostatitis alleviation by inhibiting the cGAS-STING pathway in sleep-deprived mice

**Fig. 7:**
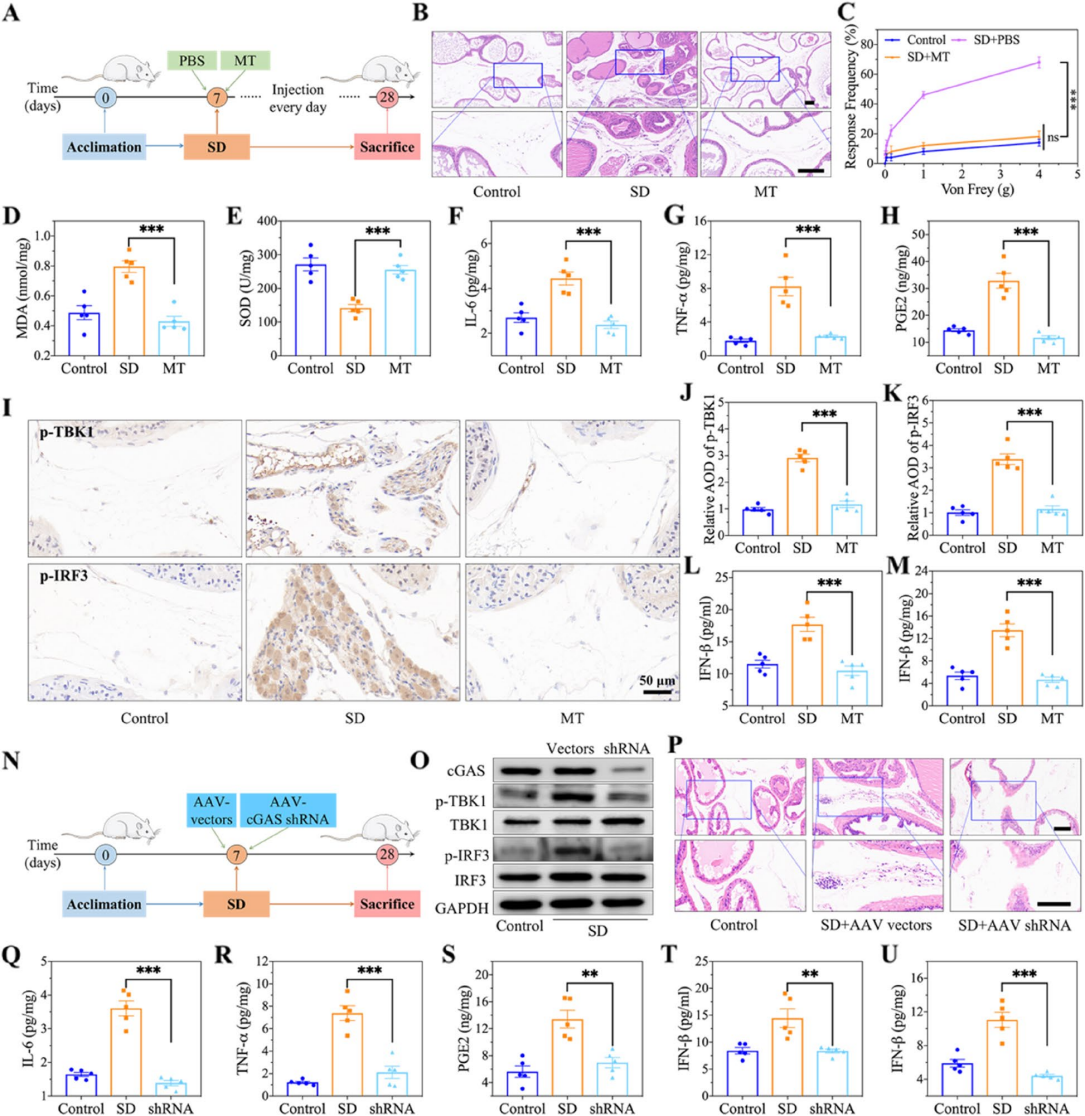
Amelioration of prostatitis by inhibiting cGAS-STING pathway in sleep-deprived mice. **A** Schematic illustration of supplement MT (4 mg/Kg) during the duration of SD in mice for 3 weeks. **B** HE staining showing the changes of prostate in mice with supplement MT after SD for 3 weeks. Scale bar, 100 μm. **C** Changes of tactile allodynia by Von Frey filaments. **D-E** The indexes of oxidative stress in prostate of mice with supplement MT after SD for 3 weeks, including MDA and SOD. **F-H** ELISA analysis of cytokines in prostate, including IL-6, TNF-α, and PGE2. **I-K** Immunohistochemical staining and corresponding AOD of p-TBK1 and p-IRF3. Scale bar, 100 μm. **L-M** The concentration of IFN-β in serum and prostate tissue. **N** Schematic illustration of the down-regulated cGAS expression by the injection of AAV-cGAS-shRNA into the prostate during the duration of SD in mice for 3 weeks. **O** Evaluation of cGAS expression in prostate of mice after 3 weeks of AAV-cGAS-shRNA injection. **P** HE staining showing the changes of prostate in mice after 3 weeks of AAV-cGAS-shRNA injection. Scale bar, 100 μm. **Q-S** ELISA analysis of cytokines in prostate, including IL-6, TNF-α, and PGE2. **T-U** The concentration of IFN-β in serum and prostate tissue.Data were presented as means ± SEM (*n* ≥ 5). Statistical significance was calculated using the one-way ANOVA (**C**, **D-H**, **J-M**, and **Q-U**). **P < 0.01, and ***P < 0.001

As mentioned above, dual-deficient MTs and DHT must be satisfied for activation of the cGAS-STING pathway. Thus, we assessed whether blocking the cGAS-STING pathway via MT supplementation would alleviate prostatitis in sleep-deprived mice (Fig. 7A). The pathological HE staining revealed fewer typical changes in prostatitis in sleep-deprived mice supplemented with MT than in those not supplemented with MT, similar to what was observed in normal mice (Fig. 7B). Likewise, pelvic pain was evaluated, and mechanical allodynia in sleep-deprived mice supplemented with MT (prevalence at 4 g via a von Frey test: 18.0 ± 3.7%) was significantly lower than that in sleep-deprived mice supplemented without MT (prevalence at 4 g via a von Frey test: 68.0 ± 3.6%) (Fig. 7C). Moreover, MT supplementation inhibited the levels of prostatic oxidative stress (MDA and SOD) and inflammatory cell cytokines, which were markedly increased in sleep-deprived mice (Fig. 7D-H), suggesting that MT supplementation can block the cGAS-STING pathway by suppressing oxidative stress. Then, we assessed the activation of the cGAS-STING pathway by immunohistochemistry, and the results showed that the expression levels of p-TBK1 and p-IRF3 in the prostatic stromal region were downregulated in MT-supplemented mice (Fig. 7I-K), suggesting that the suppression of the cGAS-STING pathway was also mediated by changes in IFN-β levels in serum and prostate tissue (Fig. 7L, M).

The aforementioned results suggested that prostatitis in sleep-deprived mice was induced via activation of the cGAS-STING pathway. To further validate this conclusion, we injected AAV-cGAS-shRNA into the prostate, downregulated cGAS expression, and subsequently induced sleep deprivation (Fig. 7N). After sleep deprivation for 3 weeks, we first measured the expression of cGAS in mice treated with AAV-cGAS-shRNA. As shown in Fig. 7O, P, the expression and downstream proteins of cGAS were inhibited, thereby suppressing the cGAS-STING pathway, which resulted in the alleviation of prostatitis in sleep-deprived mice. Additionally, compared with those in sleep-deprived mice treated with AAV vectors, the prostate tissue levels of IL-6, TNF-ɑ, PGE2 and IFN-β were lower in sleep-deprived mice injected with AAV-cGAS-shRNA; they were close to the normal range (Fig. 7Q-U). These findings adequately indicate that activation of the cGAS-STING pathway plays a vital role in mediating prostatitis in sleep-deprived mice.

### Prostatitis progression in sleep-deprived mice after recovery sleep

**Fig. 8:**
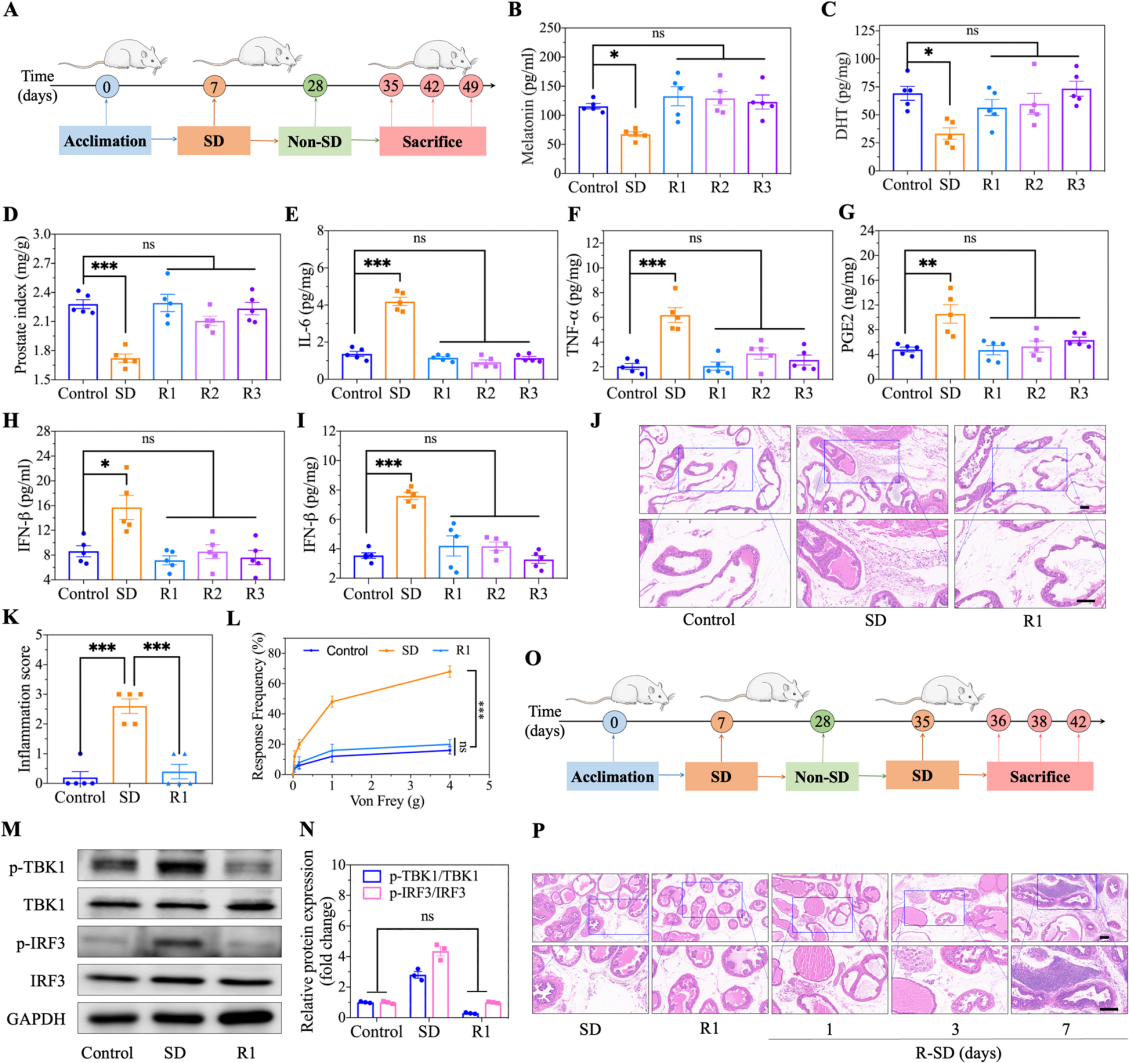
Progression of prostatitis in sleep-deprived mice after sleep recovery. **A** Schematic illustration of sleep recovery for different time (1 to 3 weeks) in SD mice. **B** Analysis of MT level in serum in SD mice after 3 weeks of sleep-recovery (R1, R2, and R3). **C** Analysis of DHT level in prostate tissue after 3 weeks of sleep-recovery. **D** The prostate sizes (prostate index) analysis. **E-G** ELISA analysis of cytokines in prostate, including IL-6, TNF-α, and PGE2. **H-I** The concentration of IFN-β in serum and prostate tissue. **J** HE staining showing the changes of prostate in SD mice after 1 week of sleep-recovery. Scale bar, 100 μm. **K** The corresponding inflammation score analysis of prostate in HE staining. **L** Evaluation of tactile allodynia by Von Frey filaments in mice. **M-N** Western blot and corresponding analysis of the essential proteins of cGAS-STING pathway in prostate of mice after 1 week of sleep-recovery. **O** Schematic illustration of sleep re-deprivation for different time (1, 3 and 7 days) in mice. **P** HE staining revealing the changes of prostate in sleep-recovery mice after sleep re-deprivation (R-SD) for different periods (1, 3, and 7 days). Scale bar, 100 μm. Data were presented as means ± SEM (*n* ≥ 5). Statistical significance was calculated using the one-way ANOVA (**B**-**I**, **K**, **L**, and **N**). ns, no significance, *P < 0.05, **P < 0.01, and ***P < 0.001

Sleep deprivation is a precipitating factor leading to MT and DHT deficiency and subsequently activating the cGAS-STING pathway. Therefore, we also investigated the progression of prostatitis in sleep-deprived mice after recovery sleep for different durations (1, 2 and 3 weeks) (Fig. 8A). We found that the expression levels of MT and DHT returned to normal levels in sleep-deprived mice after freely sleeping for a week (Fig. 8B, C). Moreover, the prostate (prostate index, 2.28 ± 0.05) returned to a normal volume (2.29 ± 0.09) and remained steady thereafter (Fig. 8D). Afterwards, we measured the levels of the inflammatory cytokines secreted by immune cells infiltrating the prostate. As a result, the expression levels of IL-6, TNF-ɑ, PGE2 and IFN-β were close to the control level and plateaued in sleep-deprived mice after one week of free sleep (Fig. 8E-I). It might be hypothesized that the inflammatory changes in the prostate in sleep-deprived mice regressed and that recovery sleep contributed to restoring the normal physiological state of the prostate. To verify our hypothesis, we utilized pathological HE staining to observe the prostate, and there were no typical changes in prostatitis in sleep-deprived mice after one week of free sleep; these findings are similar to what has been observed in normal tissue (Fig. 8J, K). Likewise, pelvic pain was evaluated by the Von Frey test, and the degree of pain in mice that had engaged in recovery sleep was significantly lower than that in sleep-deprived mice (Fig. 8L). The activation of the cGAS-STING pathway in sleep-deprived mice was obviously inhibited by recovery sleep, which has significant implications for improving prostatitis (Fig. 8M, N). These results all proved that prostatitis induced by sleep deprivation tended to resolve after one week of freely sleeping. Additionally, for mice that were sleep-deprived but that then had one week of recovery sleep, we further investigated changes in the prostate that returned to a normal physiological status after 1, 3 and 7 days of sleep deprivation (Fig. 8O). Interestingly, the degree of pathological changes associated with prostatitis became more severe with increasing sleep re-deprivation time (Figs. 8P and S6). After 7 days of sleep re-deprivation, compared with those in sleep-deprived mice, the blood vessels in sleep re-deprived mice exhibited increased density and dilation, and the infiltration of inflammatory cells around the blood vessels also increased markedly in the prostate tissue. In parallel, we noted that the intensity of pelvic pain induced by prostatitis immediately increased (Fig. S7). These results not only suggest that prostatitis induced by sleep deprivation can be cured after individuals resume sleep but also illustrate that inflammation can reappear and become increasingly severe after re-deprivation.

## Discussion

The pathogenesis and treatment strategy of prostatitis have always been complex problems for urological surgeons. Although previous studies have shown that autoimmune imbalance, neuroendocrine abnormalities, urinary reflux, urinary microbial infection and insufficient sleep may be the predisposing causes of prostatitis, the detailed and exact pathogenesis of prostatitis still needs to be clearly elucidated [36–40]. Moreover, several studies have focused on the relationship between sleep disorders and lower urinary tract symptoms and demonstrated that sleep disturbance is significantly associated with lower urinary tract symptoms, the main symptom of prostatitis [41, 42]. In addition, sleep deprivation in mice can induce conjunctivitis and pneumonia and alter ventral prostate morphology, leading to glandular atrophy, which further verified that sleep is closely related to prostatitis [43]. Therefore, this study comprehensively evaluated the association between sleep deprivation and prostatitis. We found decreased expression levels of MT and DHT in sleep-deprived mice and demonstrated that only a simultaneous deficiency of MT and DHT is necessary for the development of prostatitis. The potential mechanisms involved in the formation of an inflammatory microenvironment were clarified in detail: The cGAS-STING pathway activated by mt-DNA triggered the recruitment of inflammatory cells into the prostate. Compared with previous studies, this study, for the first time, elucidated the exact risk factors involved and the detailed underlying mechanisms involved in the development of prostatitis, identifying a potent therapeutic target for the clinical treatment of this disease.

Melatonin is one of the most powerful antioxidants and radical scavengers and can maintain the dynamic balance of free radicals and effectively protect critical molecules from ROS-mediated damage [44]. Additionally, melatonin targets mitochondria to enhance their function, maintaining mitochondrial integrity and leading to reduced electron leakage and mitochondrial ROS generation [45]. Previous studies have shown that sleep deprivation-mediated circadian rhythm disruption reduces the expression level of melatonin, leading to cell damage and inflammation, such as neuroinflammation, atherosclerosis, and vascular inflammation [46, 47]. In addition to inhibiting melatonin function (content reduction), in the present study, we found that a decreased DHT concentration was involved in the progression of prostatitis, which was different from the findings of previous studies. DHT is the most crucial hormone for prostate growth and development, and DHT depletion can cause atrophy of the prostate gland and interfere with cellular oxidative metabolism [48]. When the respiratory chain in mitochondria is disrupted, a large amount of ROS and another superoxide are produced [49]. Moreover, melatonin, a powerful antioxidant, can remove harmful ROS and protect cells from ROS-induced damage [50]. However, in sleep-deprived mice, decreased DHT and melatonin levels resulted in an imbalance in ROS generation and removal, leading to the rapid accumulation of ROS and causing ROS-mediated cell responses and damage. In addition, we also verified that prostate damage and inflammation occurred only in mice treated with both an MT receptor antagonist (4-P-PDOT) and a 5α-reductase inhibitor (finasteride). This damaging effect of the double deficiency of melatonin and DHT was presented for the first time in this study; this novel finding can elucidate the specificity of sleep deprivation-induced prostatitis and provide new ideas for prostatitis prevention and treatment. The growth of prostate was suppressed (the decline of prostate volume) through the inhibiting of DHT levels by finasteride. However, when the MT inhibitor (4-P-PDOT) was utilized to inhibit the function of MT, we did not observe any changes in DHT level and prostate volume, indicating that there is no direct regulatory relationship between DHT and MT. In addition, the main source of DHT in prostate is conversion from testosterone via type 2 of 5α-reductase (SRD5A2). The decreased levels of SRD5A2 in prostate were found, which may be a key reason for the decrease of DHT in the prostate of sleep-deprived mice. Therefore, we speculate that there is no direct regulatory relationship between MT and DHT levels, and the change of DHT in prostate may have tissue specificity and be regulated by other underlying mechanisms, which needs to be explored in the future.

In addition, we further explored the detailed molecular mechanisms that mediate inflammation after MT and DHT deficiency. In the present study, the accumulation of ROS generated by MT and DHT deficiency induced mitochondrial damage, resulting in the leakage of mt-DNA into the cytoplasm. The release of mt-DNA into the cytosol has emerged as a prominent trigger of cGAS-STING pathway activation, during which inflammatory factors are continuously produced to shape an inflammatory microenvironment [51, 52]. Therefore, cGAS-STING may be involved in the induction and regulation of prostatitis progression. Based on the above inference, through prostate protein sequencing and KEGG pathway analysis, we found that the enriched signalling pathways were significantly associated with the cGAS-STING pathway, which was verified by western blotting. Our findings were similar to the results of other studies that focused on the function of cGAS-STING in regulating other inflammatory diseases, such as neuroinflammation, atherosclerosis, vascular inflammation and enteritis [53–56]. However, the initial factors that activate the cGAS-STING pathway differ from those involved in inflammation in other organs reported in previous studies. One study focused on the effect of melatonin on intestinal mucosal injury and microbiota dysbiosis in sleep-deprived mice [57]. The results showed that the effect of sleep deprivation on intestinal barrier dysfunction might be an outcome of melatonin suppression, highlighting the pathogenicity of melatonin reduction. In the present study, the development of prostatitis was based on the ROS accumulation caused by DHT reduction and decreased ROS scavenging ability due to MT depletion rather than solely through melatonin depletion, which highlighted that prostatitis induced by sleep deprivation has a specific regulatory mechanism. Therefore, inhibiting the cGAS-STING signalling pathway and increasing MT and DHT levels are potential strategies for treating prostatitis induced by sleep deprivation. We used cGAS knockdown and MT supplementation to inhibit the cGAS-STING pathway in sleep-deprived mice, and the results showed that prostatitis was not induced during the same period. This indicated that MT can be solely used for the treatment and prevention of prostatitis, which is consistent with our team’s previous research [15]. Thus, our findings could not only guide the prevention of sleep deficiency-related prostatitis but could also provide promising treatment strategies for patients with prostatitis.

Additionally, several studies have shown that sleep deprivation is correlated with prostatitis-related lower urinary tract symptoms, and improving sleep quality (total sleep time) is significantly helpful for alleviating lower urinary tract symptoms, which is consistent with our findings [58]. In this study, after 1 week of recovery sleep, we found that inflammation in the prostate of sleep-deprived mice was resolved, as characterized by successful clearance of hyperemia, dilatation of blood vessels and perivascular infiltration of inflammatory cells. However, when sleep-deprived mice that had engaged in recovery sleep for one week were redeprived of sleep for seven days, a large amount of infiltrated inflammatory cells and fibrosis appeared in the prostatic stroma, which suggested that sleep deprivation induced prostatitis faster and more robustly in the newly recovered mice. In the clinic, prostatitis is prone to recurrence, and treatment failure is common [59, 60]. Therefore, our findings could provide new insights into recurrent prostatitis in the clinic, as well as new strategies for the further treatment of prostatitis. From our point of view, eliminating the causative factors for prostatitis is more critical than medication for prostatitis and requires further investigation and verification in the clinic.

## Conclusions

In conclusion, the present study provides evidence that sleep deprivation, as an initial pathological trigger, promotes cGAS-STING pathway activation through dual deficiency of MT and DHT. The activated cGAS-STING pathway then recruits inflammatory cells into the prostatic stroma via the secretion of cytokines (IFN-β), thereby causing the development of prostatitis. Together, our findings will offer new insights into the occurrence of clinical prostatitis and provide potential therapeutic targets and strategies.

### Supplementary Information

**Supplementary Material 1.**

## Data Availability

No datasets were generated or analysed during the current study.

## Abbreviations

SD: Sleep deprivation
DHT: Dihydrotestosterone
ROS: Reactive oxygen species
mt-DNA: Mitochondrial DNA
MT: Melatonin
T: Testosterone
E2: Estradiol
LH: Luteinizing hormone
GnRH: Gonadotropin-releasing hormone
FT: Finasteride
ds-DNA: Double-stranded DNA
p-TBK1: Phosphorylation of TBK1
p-IRF3: Phosphorylation of IRF3
AOD: Average optical density
CP/CPPS: Chronic prostatitis/chronic pelvic pain syndrome
