# Supplementary material for: Dual deficiency of melatonin and dihydrotestosterone promotes stromal cell damage and mediates prostatitis via the cGAS-STING pathway in sleep-deprived mice
Source: Cell Commun Signal. 2024 Mar 15;22:183. doi: 10.1186/s12964-024-01554-5 (PMC10941623; doi:10.1186/s12964-024-01554-5)
Supplement: Supplementary file 1 — Supplementary Material 1. [file 12964_2024_1554_MOESM1_ESM.docx]

**Supporting Information for**

**Dual deficiency of melatonin and dihydrotestosterone promotes stromal cell damage and mediates prostatitis via the cGAS-STING pathway in sleep-deprived mice**

Jia Chen^1^, Wenming Ma^1^, Shaoyu Yue^1^, Dongsheng Li, Lei Chen, Cheng Zhang, Yu Guan, Chun Li, Changqin Jiang, Guiyi Liao, Chaozhao Liang^*^, Hui Wang^*^, Sheng Tai^*^

Department of Urology, the First Affiliated Hospital of Anhui Medical University

Institute of Urology, Anhui Medical University

Anhui Province Key Laboratory of Genitourinary Diseases, Anhui Medical University, Hefei 230022, P.R. China

^1^These authors contributed equally to this work.

^*^Corresponding author:

Chaozhao Liang, PhD, Email: [liang_chaozhao@ahmu.edu.cn](mailto:liang_chaozhao@ahmu.edu.cn); Hui Wang, PhD, Email: [whayd@sina.cn](mailto:whayd@sina.cn); Sheng Tai, PhD, Email: [taisheng@ahmu.edu.cn](mailto:taisheng@ahmu.edu.cn)


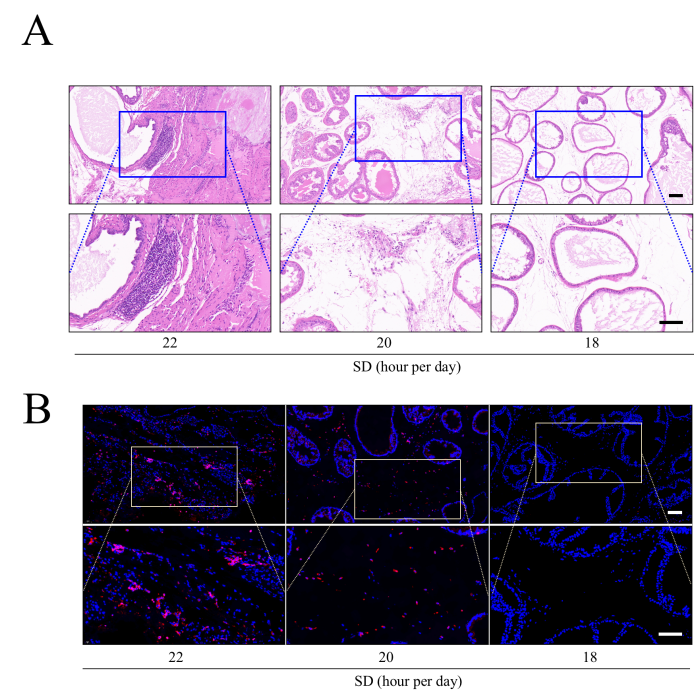


**Fig. S1** **A** HE staining and **B** CD45 immunofluorescence staining showing the infiltration of inflammatory cells in the prostatic stroma of mice after different SD time per day for 4 weeks. Scale bars, 100 μm.


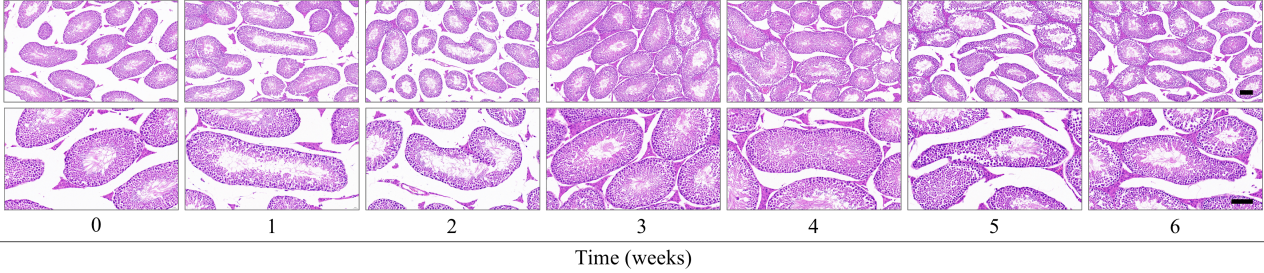


**Fig. S2** Histopathological examination of testicles in mice after different SD duration (0 to 6 weeks). Scale bars, 100 μm.


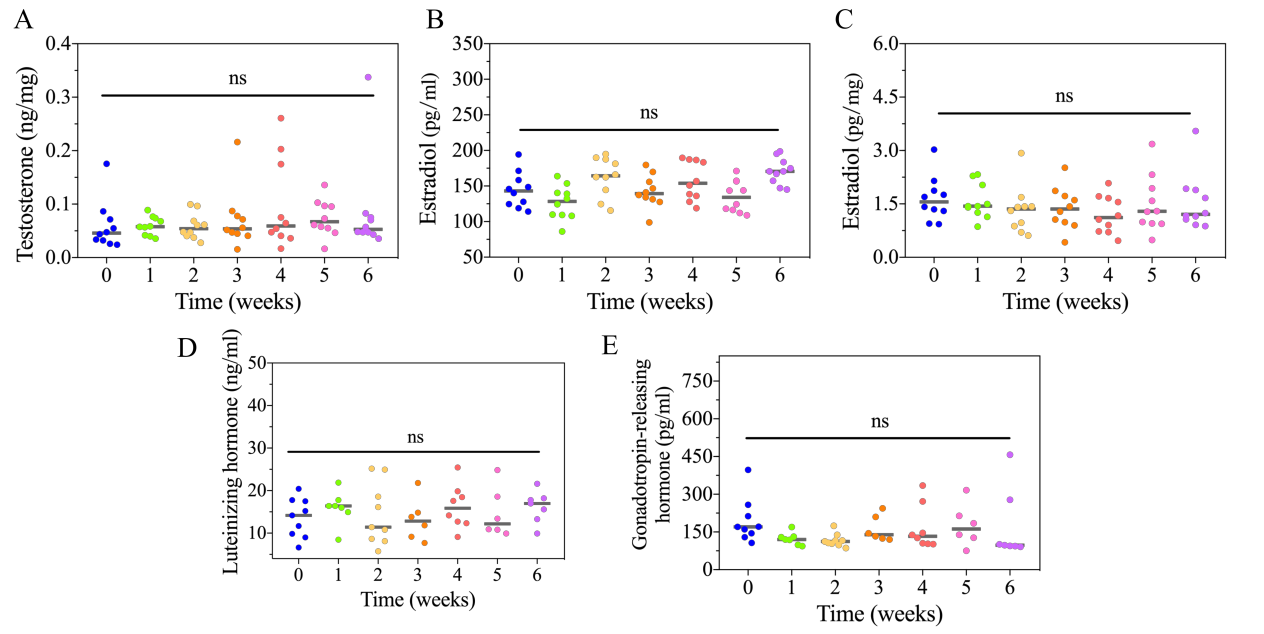


**Fig. S3** ELISA analysis of **A** testosterone level in prostatic tissue, estradiol level in **B** serum and **C** prostatic tissue, **D** LH and **E** GnRH level in serum for mice with different SD duration (0 to 6 weeks) (n ≥ 6). Data were presented as means ± SEM. Statistical significance was calculated using the one-way ANOVA. ns, no significance.


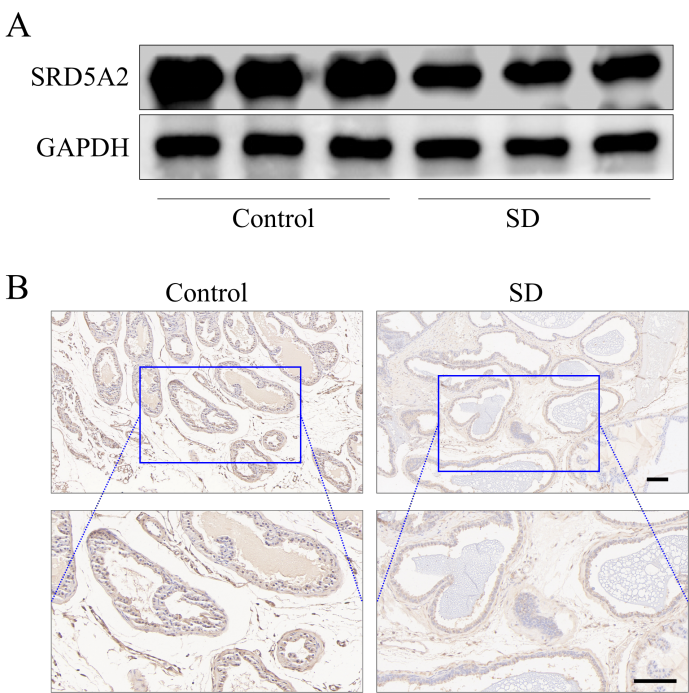


**Fig. S4** **A** Western blot and **B** immunohistochemical staining showing the expression changes of SRD5A2 in prostate of mice after SD for 3 weeks.


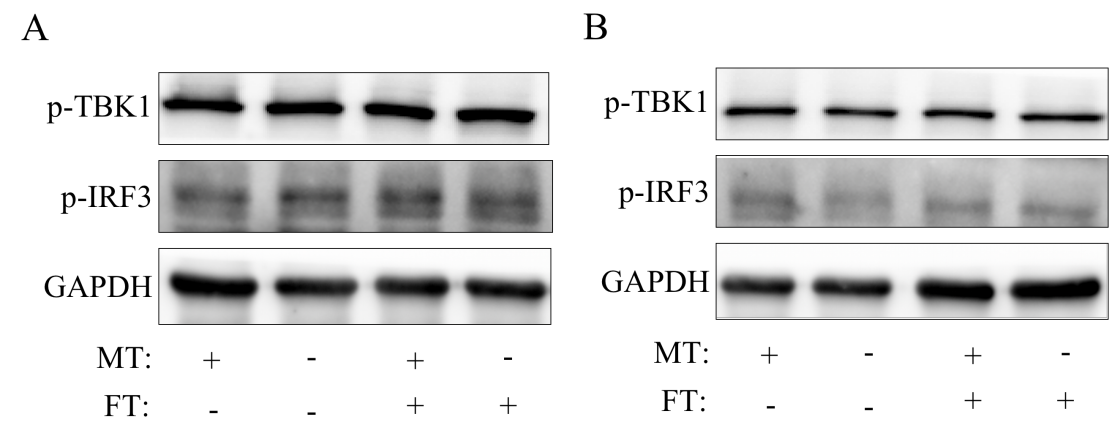


**Fig. S5** The expression changes of p-TBK1 and p-IRF3 in prostate epithelial cells (RWPE-1 cells) after treated with different concentrations of MT and FT for 1 **A** day and **B** 3 days.


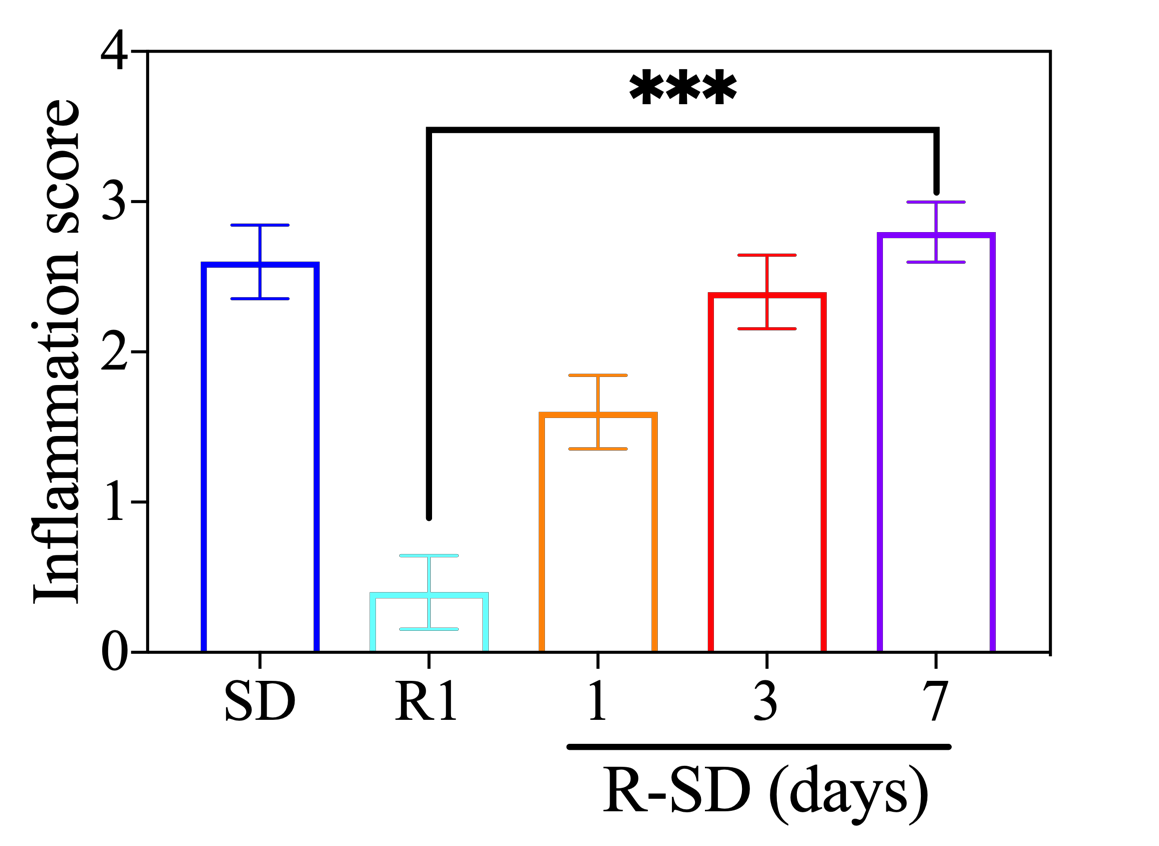


**Fig. S6** Inflammation score in prostate of mice after 1 to 7 days of sleep re-deprivation (n ≥ 3). Data were presented as means ± SEM. Statistical significance was calculated using the one-way ANOVA. ***P < 0.001.


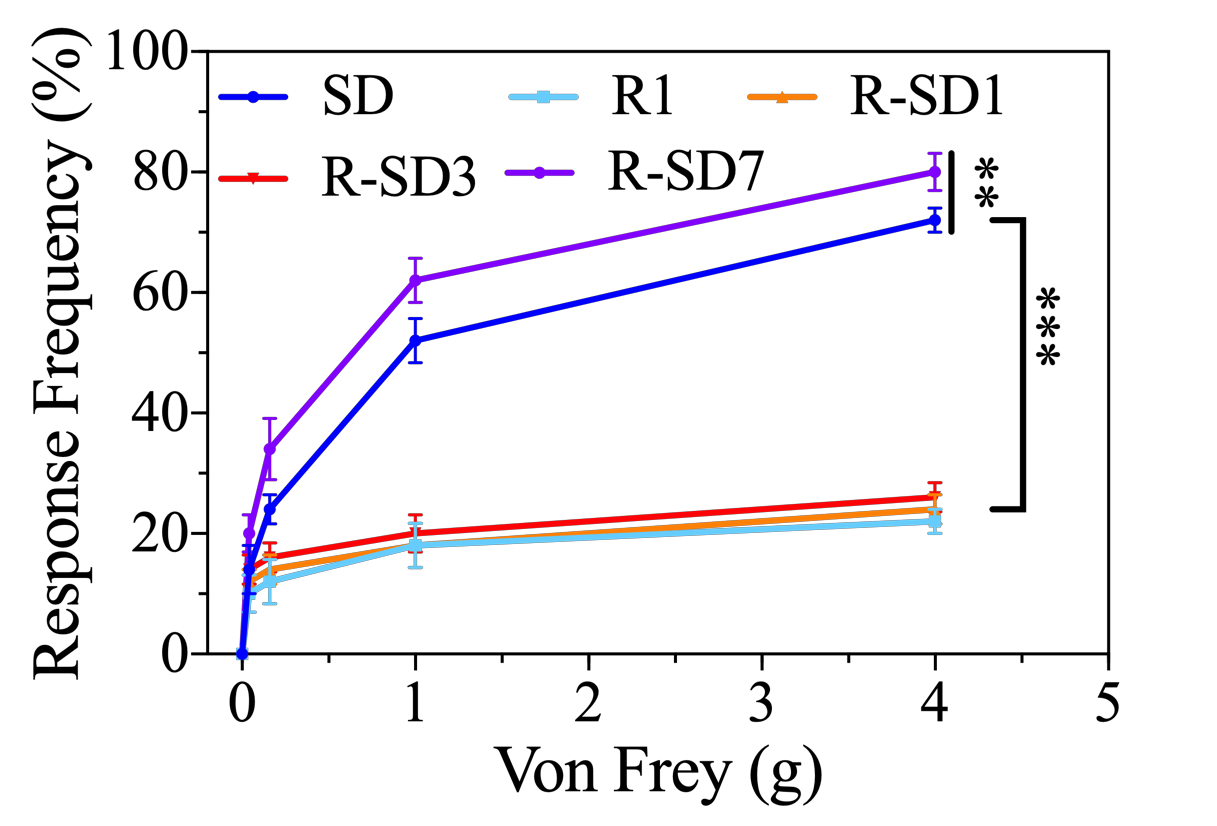


**Fig. S7** Tactile allodynia analysis in prostate of mice after 1 to 7 days of sleep re-deprivation (n ≥ 3). Data were presented as means ± SEM. Statistical significance was calculated using the one-way ANOVA. **P < 0.01 and ***P < 0.001.

**Fig. S8** The original western blot images corresponding to Fig. 4I. M (Marker), prestained protein ladder.

**Fig. S9** The original western blot images corresponding to Fig. 4L. M (Marker), prestained protein ladder.

**Fig. S10** The original western blot images corresponding to Fig. 5I. M (Marker), prestained protein ladder.

**Fig. S11** The original western blot images corresponding to Fig. 7O. M (Marker), prestained protein ladder.

**Fig. S12** The original western blot images corresponding to Fig. 8M. M (Marker), prestained protein ladder.

**Fig. S13** The original western blot images corresponding to Fig. S4. M (Marker), prestained protein ladder.

**Fig. S14** The original western blot images corresponding to Fig. S5A. M (Marker), prestained protein ladder.
